# Peripheral Network Connectivity Analyses for the Real-Time Tracking of Coupled Bodies in Motion

**DOI:** 10.3390/s18093117

**Published:** 2018-09-15

**Authors:** Vilelmini Kalampratsidou, Elizabeth B. Torres

**Affiliations:** Psychology Department, Center for Biomedicine Imaging and Modeling, Computer Science Department, Rutgers Center for Cognitive Science, Rutgers University, New Brunswick, NJ 08854, USA; vilelmini.kalabratsidou@gmail.com

**Keywords:** sensor grids, weighted directed graphs, network connectivity, dyadic coordination, cohesiveness, spontaneous synergies, motor reciprocity, stochastic signatures, ballet partnering

## Abstract

Dyadic interactions are ubiquitous in our lives, yet they are highly challenging to study. Many subtle aspects of coupled bodily dynamics continuously unfolding during such exchanges have not been empirically parameterized. As such, we have no formal statistical methods to describe the spontaneously self-emerging coordinating synergies within each actor’s body and across the dyad. Such cohesive motion patterns self-emerge and dissolve largely beneath the awareness of the actors and the observers. Consequently, hand coding methods may miss latent aspects of the phenomena. The present paper addresses this gap and provides new methods to quantify the moment-by-moment evolution of self-emerging cohesiveness during highly complex ballet routines. We use weighted directed graphs to represent the dyads as dynamically coupled networks unfolding in real-time, with activities captured by a grid of wearable sensors distributed across the dancers’ bodies. We introduce new visualization tools, signal parameterizations, and a statistical platform that integrates connectivity metrics with stochastic analyses to automatically detect coordination patterns and self-emerging cohesive coupling as they unfold in real-time. Potential applications of these new techniques are discussed in the context of personalized medicine, basic research, and the performing arts.

But there’s nothing more profound than creating something out of nothing.

—Rainbow Rowell

## 1. Introduction

Successful human social exchange relies on properly coupling the dynamics of biorhythms embedded in speech, facial expressions, gaze, gestures, and body postures. While research involving speech and face-processing techniques in the context of dyadic exchange has been fruitful [1,2], there is a paucity of full-body models that allow the continuous tracking of self-emerging cohesiveness dynamically *shifting in real-time*, as synchronous patterns emerge and dissolve during continuous physical dyadic exchange.

The real-time tracking of shifts in stochastic signatures of biophysical signals co-registered with commercially available and high-grade biosensors is important to begin the path of promoting closed-loop biofeedback-based therapies. Such families of therapies can be guided by stochastic shifts in the biorhythm fluctuations, unique to each person [3,4]. Indeed, a personalized approach to fluctuations from voluntary movements [5] is amenable to developing clinical interventions that are individually tailored to the person’s nervous system sensory capabilities and predispositions [6,7]. For example, in disorders of the nervous systems that are highly heterogeneous in nature (autism, Parkinson’s disease, schizophrenia, etc.), such individualized approaches are bound to be more effective than using a “*one size fits all*” model based on grand averages of biophysical signals using population statistics [8].

Personalized approaches can be used within the closed-loop stochastically guided feedback control. To that end, we have adapted the principle of reafference (by von Holst and Mittelstaedt [9,10] Chapter 1) in the context of dyadic coupled behaviors. This principle states that “*Voluntary movements show themselves to be dependent on the returning stream of afference which they themselves cause*”. Within this theoretical framework, we define the returning stream of voluntary motions as consequential motions, to begin the path of understanding causality in natural dyadic behaviors. As such, we have empirically discovered a reclassification of kinesthetic reafference according to their stochastic features, whereby we clearly divide actions into those that are deliberate (dynamics-invariant) and those that are consequential (dynamics-covariant) [5,11,12,13,14].

The combination of kinesthetic reafference in closed-loop interfaces with stochastic parameterization of the moment-by-moment motor fluctuations, has helped us evoke volitional control and enhance motor autonomy in non-verbal children with autism of a high severity [6]. In such naturalistic settings, the use of consequential motions in sensory substitution/augmentation interventions has helped dampen the excess random noise in the autistic nervous systems [7,15] and defined proof of concept for a new generation of closed-loop therapies grounded on the consequential motor-sensing concept [3,10]. Since such therapies often require dyadic exchange between the child and the therapist, we reasoned that it would be necessary to develop new methods amenable for real-time forecasting and detection of the type of cohesiveness that spontaneously emerges from coupled behaviors. As such, we have developed new standardized data types and analytics that permit real-time tracking of key aspects of the interaction. To test such methods, in this study, we use complex ballet partnering as our testbed, and probe aspects of motor reciprocity, physical entrainment, synergies, and synchronicity that have been researched by other fields using different methods.

The study of entrainment and spontaneously emerging coordinating behaviors between two interactive actors in the social realm has been previously researched in ecological psychology [16,17,18,19,20,21]. This field of study has a growing body of work devoted to the evaluation of intra- and inter-personal exchange, with a focus on the coordination and synchrony of specific body parts [22] and between two people [19]. The pioneering work has often focused on prescribed laboratory tasks, or on naturalistic tasks [23,24] that are nonetheless examined under a priori parametric assumptions of the underlying random process that the motion parameters may describe. As such, under the general framework of statistical hypothesis testing, the extant literature on intra- and inter-personal dyadic exchange has been more focused on summary statistics of the overall interaction, approached a posteriori, rather than on the natural unfolding dynamics of the social exchange.

In its current form, the traditional approach does not offer the possibility of examining complex shifting dynamics of cohesiveness in dyadic behaviors under *empirically estimated* families of probability distributions. Such families may be used to represent the evolution of motor synergies unfolding *in real-time*. Indeed, recent studies of dyadic social exchange in the context of autism diagnoses point at the importance of considering the shifting of empirically estimated stochastic signatures characterizing the biophysical signals of coupled bodies in motion [3,25] (see Appendix A), rather than assuming a general-population theoretical model (as explained in [8]).

**Figure 1:**
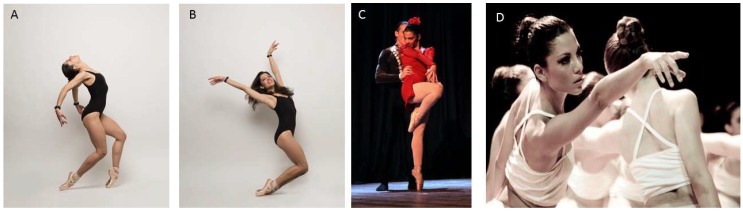
Coordination of complex patterns of behavior in multiple settings. (**A**,**B**) Maintaining and controlling difficult postures. (**C**) Building synchronous synergies in dyadic exchange. (**D**) Maintaining a harmonious flow in a crowd of dancers performing a choreography.

The type of empirical work that we bring to the reader’s consideration is important to begin the path of *empirical parameterization* of the statistical signatures (and their real-time shifts) of the types of consequential motions that complement voluntary, goal-directed behavior and that ultimately contribute to autonomous control. These different movement classes have been precisely characterized individually in recent work involving complex boxing routines [5,6,26] and routines from the performing arts [27]. Coordination patterns of synergistic modules self-emerge as the person’s body sustains complex postures up against gravity (Figure 1A,B). They can be physically registered and directly obtained through various sensors. However, they are more challenging to study in dyadic exchange (Figure 1C), or as a group of actors interacts to form fluid choreographic patterns (Figure 1D), owing to the transient and dynamically changing nature of the type of cohesiveness that spontaneously self-emerges from the two bodies in motion. This type of activity is not physically registered but rather synthetized by the physical entrainment of the two moving bodies. Current approaches to dyadic exchange often assume stationary models and consequently have not addressed the types of non-stationary signals that we bring to the reader’s attention.

In this paper, we develop a general platform for analyses of time-series of biophysical signals. This platform introduces new parameterizations of the bio signals and stochastic methods to study dyadic interactions. The methods are adaptable to different biosensors and motion caption systems. In the main paper, we introduce the methods and illustrate their use with an optical motion caption system with a high sampling resolution; while in the Appendix B, we show the use of the methods integrating grids of wearable inertial measurement units (IMUs) with a sampling resolution comparable to commercially available IMUs. We test these for partnering dancers and clinical settings. In all cases, we empirically parameterize fluctuations in spontaneous cohesiveness in new ways amenable to enable the *real-time tracking* of patterns of variability in physical dyadic exchange. Within the context of partnering dances, the methods track complex synergies in each dancer and coupled bodily and postural motion coordination dynamics between the two participants of the dyad, as their complex dances naturally unfold *in real-time*.

## 2. Materials and Methods

### 2.1. Motivation: Deliberate vs. Consequential Motions Self-Generated by the Nervous Systems

There is a facet of complex human movements that transpires largely beneath awareness, as the person engages in voluntary movements. We have been able to capture and model such hidden segments of behavior in sports and the performing arts, and characterize their stochastic signatures as the athlete (or the performing artist [27]) trains and adapts to new routines [5,28]. These latent movement segments, which we have coined consequential to voluntary segments, have the fundamental feature that they change the geometry of the hand motion trajectory under different dynamic manipulations involving changes in speed and body mass distributions [5,11,12,13,14]. Further, as complex sport routines unfold, motor variability automatically separates these consequential segments from those deliberately aimed at a goal [6,28,29]. In contrast, the geometric features of deliberate hand trajectories remain steady, despite changes in dynamics (see also [3] Chapter 4).

Such deliberate-consequential dichotomy more generally extends from the hand (the end effector) to trajectories of complex full-body motions generated by one subject. These include those performed by non-human [13,14] and human subjects [5,12,13,14,30,31]. We ask if the distinction between deliberate and consequential modes of action would also extend to dyadic behaviors. This work parameterizes the stochastic signatures of spontaneously self-emerging cohesiveness of ballet partnering, as shifts in cohesiveness fluctuate during deliberately rehearsed motions and during spontaneously improvised behaviors of intermezzo and calibration periods. We ask if rehearsed vs. improvised motions can be statistically distinguished (despite both being composed of deliberate and consequential segments).

### 2.2. Data Acquisition and Signal Processing

The data for this project was kindly donated to our lab by the Phase Space team. The work was part of an effort to merge aspects of the performing arts and robotics technology by Tarik Abdel-Gawad formerly of Bot & Dolly (currently at Google) and Kan Anant of the Phase Space. The set-up can be seen in Video S1.

The rehearsed dance pieces include segments of independent dancing (dance different choreographies without making any physical contact), synchronized dancing (dance same choreography without making any physical contact), and interactive dancing (dance affecting each other, e.g., get or give support to the other person, push, pull, and lift each other, among others). We pool these rehearsed segments and coin them “*the dancing condition*”. They require timely coordination and synchronous (synergistic) activities between the two dancers. We assess the variability patterns of each of the 14 rehearsed dancing segments and empirically estimate their stochastic signatures. These segments’ raw data can be seen in Table S1. We processed the data to clean it of missing segments due to occlusions and used the continuous trajectories in our analyses.

In addition to the actual rehearsed choreography, the recordings also contain movements of taking the T-pose to set-up and calibrate the motion capture system. These also included walking to the initial pose and spontaneously improvising or reviewing/planning aspects of the choreography. These sections were named “*the non-dancing condition*”. They are not structured as the rehearsed ones, so they offer the opportunity to compare motions that are rehearsed and generally aimed at a choreographic goal and motions that are not rehearsed and do not have an overarching well-defined goal. Although both classes of routines contain deliberate and consequential segments, one is clearly less structured than the other and as such, may afford more variability. Each recorded segment piece lasted from 0.5 to 2.1 min. Pooling across sub-routines gave us 23 min of kinematics data for each condition recorded at 960 Hz split into 480 Hz per dancer (see below).

### 2.3. Instrumentation Specs

**Figure 2:**
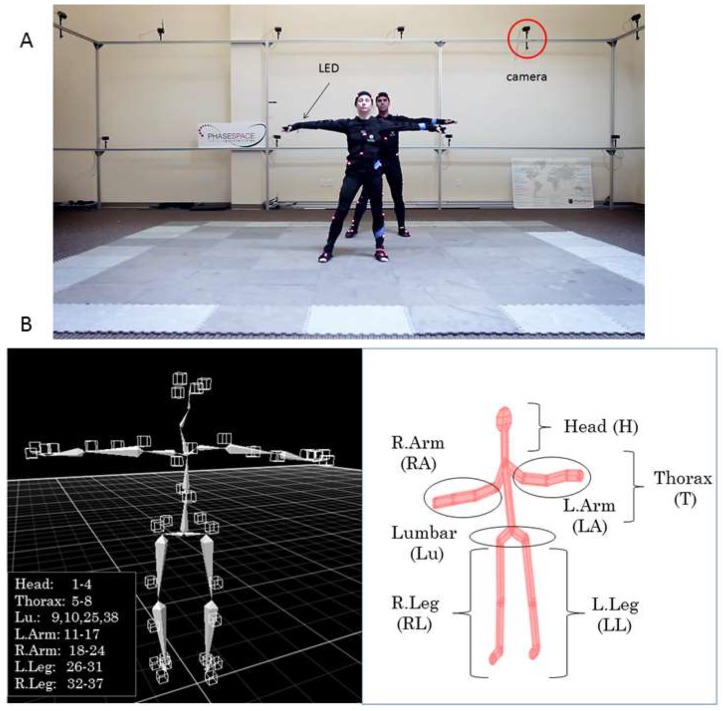
The data collection and representation tools. (**A**) Two professional dancers in T-pose while being calibrated within the Phase Space. Cameras capturing the motion are marked and suits contain 38 LEDs in each dancer’s body. Data is sampled at 960 Hz. (**B**) (**Left**) panel: Skeleton showing the distribution of LEDs from 1–38 across the body segments. (**Right**) panel: Our avatar designed in Matlab using a forward kinematics model in [32] to track the various parameters of interest (see movie from the Phase Space and Bot and Dolly in Link 1 of the Appendix B.).

Movements were recorded using a 24 camera Impulse X2 Phase Space Motion Capture system (960 Hz, San Leandro, CA, USA) (Figure 2A). A total of 76 active Light Emitting Diodes (LED) sensors were utilized and mounted on a suit across the body (Figure 2B). Each dancer had 38 sensors spread across the body: 4 on the head, 12 on the legs (2 × 6, on each leg), 14 on the arms (2 × 7, in each arm), 4 around the pelvis (lumbar and hip areas), and 4 around the thorax. The left panel of Figure 2B shows the sensors’ location on the skeletal representation, while the right panel of Figure 2B shows the avatar representation we designed using a forward kinematics map, to capture our movies and represent our data types.

### 2.4. Pre-Processing

**Figure 3:**
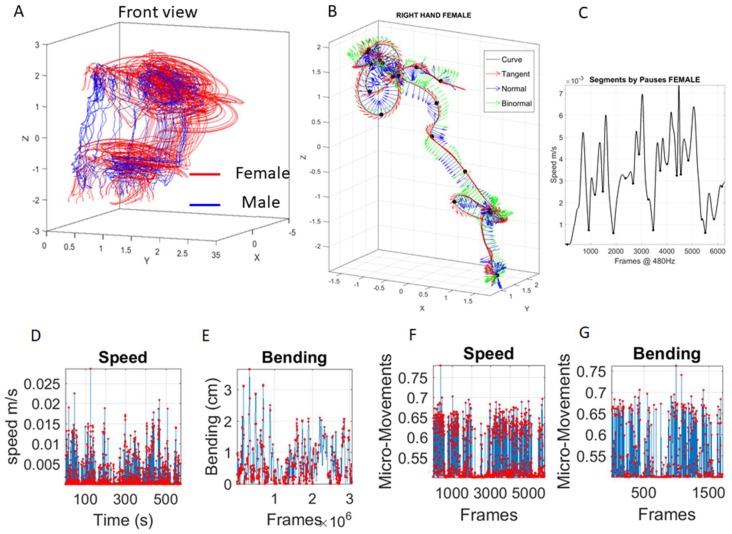
Building our new micro-movements data type from kinematic parameters extractable from positional movement trajectories. (**A**) Two views of the movement trajectories from the two dancers (red female and blue male) while performing one segment of a dance routine. (**B**) Sample trajectory from the female hand during one routine. Frenet-Serret frames along the trajectory to compute curvature and torsion parameters. (**C**) Linear speed profile of the hand trajectory segment in (**B**) to automatically extract the pauses from the speed’s local minima marked with dots (corresponding to the dots on the three-dimensional trajectory of (**B**)). (**D**) Speed profile of one LED sensor across 500 s of motion and (**E**) corresponding trajectory bending profile. (**F**) Micro-movements scaling linear speed profile in (**D**) and bending profile in (**E**) to obtain the standardized wave form representing a continuous random process as spikes in signals’ amplitude fluctuations depicted in **F**–**G**.

Positional data from each of the 76 sensors were examined. Figure 3A shows sample trajectories of the two dancers for a snippet of a routine. Figure 3B focuses on one sample sensor from the right hand of the female. The Frenet-Serret frame [33] and the speed profile (Figure 3C) are used to study geometric parameters (curvature, torsion, speed curve, speed acceleration, path length, etc.). Further, the local minima of the time series of speed profiles derived from the positional trajectories are used to automatically separate the segments of the trajectory (Figure 3C local minima correspond to pauses in Figure 3B along the female hand’s trajectory).

Using the curvature of the trajectory we derived the amount of epsilon-local bending of the curve (with no self-interceptions (Self-intersections can be dealt with torsion (twisting of the curve) but their profiling is beyond the scope of the biometrics we present here, as we focus on the planar aspects of the curves through the bending metric.)) relative to the straight-line segment connecting two sampled points by measuring the distance from the point on the curve to the point on the local line at a right angle. The bending profile for the segment (resampled frames) is shown in Figure 3E (in cm), next to the linear speed profile of that segment shown in Figure 3D (in cm/s).

### 2.5. First Parameterization: The Micro-Movements

The bending (and the speed) profiles provide a continuous waveform that we re-parameterize as spikes (events in time) describing fluctuations in amplitude (peaks) of the parameter of interest (e.g., bending or linear speed, etc.). Unlike cortical spikes, which are binary, this scaled version of the raw waveform ranges between 0 and 1 in the real domain. To convert the bending or the speed profiles to such (analogue) spike trains, we use our data type coined micro-movements (MM).

These MM track the fluctuations in the amplitude and/or peak timings of the original waveform and scale them to account for anatomical differences. Figure 3F,G shows an example from the female’s right hand marker trajectory (in Figure 3B), using, in this case, the bending profile obtained over 10,000 frames and extracted scaled micro-movements (ranging between 0 and 1).

**Figure 4:**
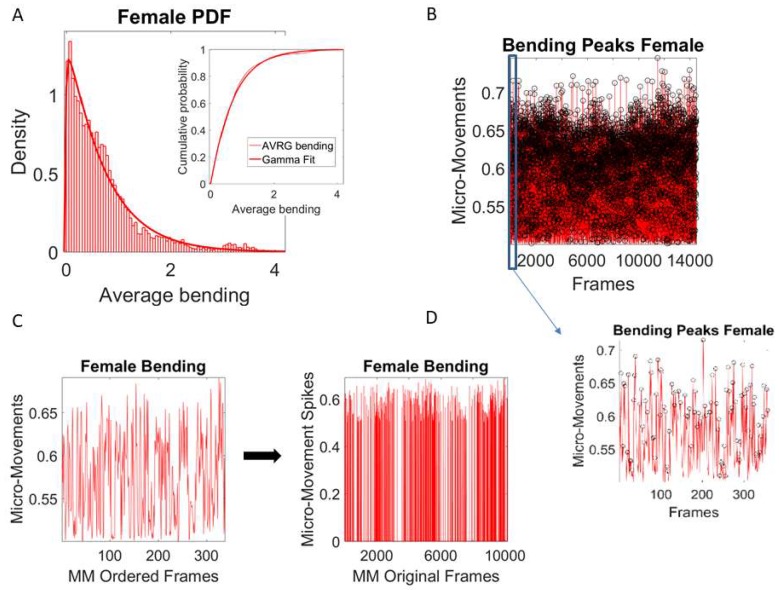
Pipeline of signal processing to obtain the micro-movement spikes of the bending amplitude as input to a Gamma process and cross-coherence analyses. (**A**) Average bending of the peaks from female dancer (one motion LED) obtained from Equation (2) of the main text is Gamma-distributed. Similarly, the peaks of the bending (not shown) are Gamma-distributed. As such, the MM derived from the normalization formula in Equation (2) are also Gamma-distributed and can be modeled by a Gamma process. (**B**) Extraction of MM from full bending profile of a routine, with inset showing the MM spikes corresponding to a segment automatically extracted from speed pauses (see Figure 3B,C). (**C**) Sampled 350 peaks (in the order in which they were acquired) from the motion path curvature generated by one LED (on the female) during a routine registered with 10 K frames are normalized as per Equation (2). They provide the amplitude MM scaled between 0–1 and are then zero-padded to recover the original number of frames in that routine segment. This procedure applied to all LEDs from both dancers then provides equal length MM spike vectors for pairwise cross-coherence analyses across all body parts of the two dancers. (**D**) Full MM spikes across all frames, zero-padded to retain equal number of frames for cross-coherence analyses.

To attain the MM that we input to the Gamma process, we mean shift the original data to center it at the empirically estimated Gamma mean value and then, for each local peak fluctuation away from the mean (taken across all frames of the bending (speed) profile of each routine segment), we produce a normalized scaled value of the peak according to Equation (1):

(1) $$Normalized\;Peak=\frac{Local\;Peak}{Local\;Peak+Local\;Average_{\min-\mathrm{to}-\min}}$$

where the peaks and the local average values computed between the local minima surrounding the peaks are also Gamma-distributed (e.g., the frequency histogram of the average bending (cm) from the female (Figure 4A) movement trajectories are plotted and fit with Gamma PDF with inset showing the Gamma CDF fit).

The unitless MM representing the real-numbered spike trains spanning between 0 and 1 then provide a standardized waveform that we adopt as our data type (the left panel of Figure 4C shows the MM waveform of one LED for a routine segment, while the right panel shows all MM occurring across all the frames of the routine segment, setting the frames where no peak was detected to 0). Figure 4D shows all frames for the entire routine with a slice of 400 frames as an inset showing the MM peaks of one subroutine. The MM data type accounts for possible allometric effects exerted by the different anatomical features of the participants [34] so we can compare individualized movement-related variations across different participants.

Each participant’s time-series (linear speed and bending) data from each body node is treated as a Gamma process (see below), and the shape and scale parameters of the continuous Gamma family of probability distributions are empirically estimated to derive the probability space of these routines for these dancers.

### 2.6. Distance Estimation in Probability Space

To ascertain similarities or differences across probability space, we use the Kantorovich/Wasserstein distance metric, also known in AI research circles as the Earth Mover’s Distance [35,36]. This distance metric is discussed in the literature in the context of Wasserstein Generative Adversarial Networks (WGANs) [37] used in optimal transport problems and proven to turn some spaces of probability measures into separable complete metric spaces [38]. This distance is appropriate as a similarity metric in probability space whereby points representing probability densities may include skewed distributions (as is the case with biophysical parameters derived from activity self-generated by human nervous systems [3]). This measure has the advantage of being a proper distance metric satisfying the triangle inequality and symmetry condition (in contrast to, e.g., the Kullback-Leiber divergence, a measure of how one probability distribution diverges from another expected probability distribution [39]). We have also tried the squared root of the Jensen-Shannon divergence [40] (i.e., the Jensen-Shannon distance metric, based off KL-divergence, with important differences), but found, based on others’ theoretical work [37] and our own work on optimization methods and gradient flow estimation for real-time computations, that the KW distance more appropriately captured the rates of change in stochastic shifts (but these are aspects of the problem beyond the scope of this paper).

**Figure 5:**
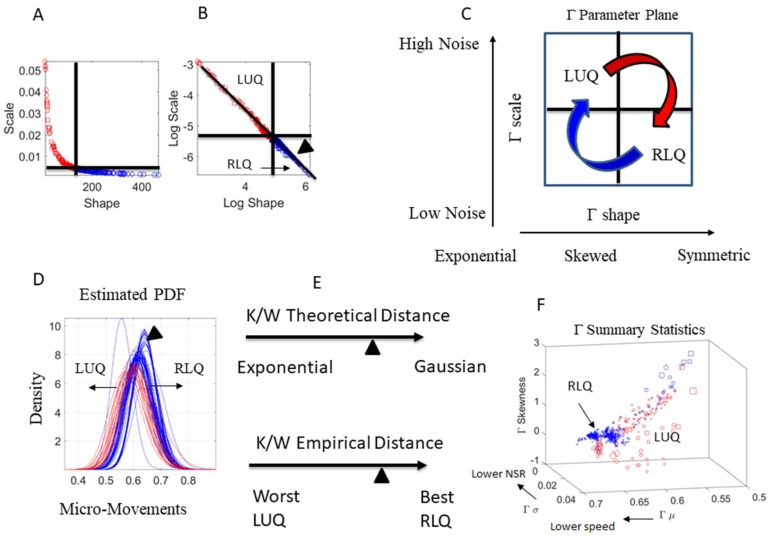
General characterization of stochastic signatures and empirically-derived criteria for targeted performance. (**A**) The Gamma parameter plane spanned by the shape and scale dimensions. Each point localizes the signatures of the person’s performance for a given segment of the routine. The quadrants defining regions of interest to track performance are defined by the median of the shape and scale parameters (dynamically changing over the routines) and empirically defining the good region by the right lower quadrant (RLQ) with probability distributions tending to the Gaussian (symmetric) with a low noise to signal ratio (low scale value). The left upper quadrant (LUQ) instead denotes regions of high randomness (towards 1, the shape value of the memoryless Exponential distribution) and with a high noise to signal ratio. (**B**) The log-log Gamma Parameter plane yields a power low-like relation of the MM data. The arrow head represents one PDF in the RLQ, localizing it in in (**D**–**F**). (**C**) Schematics representing the different stochastic states of the data, as they evolve over time. (**D**) Empirically estimated Probability Density Functions (PDFs) from the data in (**A**,**B**). (**E**) Different distance criteria to ascertain similarities and differences between points in the probability space of interest. One criterium uses theoretical limiting points (Exponential with high noise vs. Gaussian with low noise), while the other uses empirically-estimated limiting points, derived from extrema of the entire data set. (**F**) The Gamma summary statistics providing the empirically-derived moments of the distributions fit. Regions of interest are color-coded as in the previous panels. Scale from 0–1 corresponds to speed MM range.

We empirically estimate each body node signature as a point in probability space. Feeding the MM spikes as input to a Gamma process, we map the estimated stochastic signatures for each dancer on the Gamma parameter plane, along the shape and scale dimensions as represented in Figure 5A, with Figure 5B showing the power law relation that emerges from the log-log Gamma parameter plane. Here, we distinguish two regions of interest delimited by the median of the shape values and the median of the scale values taken across all body nodes and routine. The empirically derived ‘good region’ is located in the right lower quadrant (RLQ) where the shape is maximally symmetric (tending to the Gaussian probability distribution function) and the noise to signal ratio is minimal. This region of goodness was determined from testing thousands of neurotypical controls ranging from 3 to 61 years of age in cross-sectional human data. It was also tested in longitudinal studies involving neonates [41]. In contrast, the ‘bad region’ is located in the left upper quadrant (LUQ) of the Gamma parameter plane, represented in Figure 5C in schematic form.

We underscore that the criteria for the best stochastic signatures (lowest noise to signal ratio and maximal symmetry) have been previously empirically determined from data involving athletes, performing artists, and typically developing individuals [5,28], in contrast to individuals with pathologies of the nervous systems (e.g., [7,26]) showing the highest noise to signal ratio and shape best fit by the memoryless Exponential distribution. The latter provides criteria for the bad region.

More specifically, the Gamma distribution family has two parameters: the shape (*a*) describing the shape of the distribution (e.g., exponential, skewed, symmetric); and the scale (*b*) describing the dispersion (high vs. low noise to signal ratio). We estimate each of these parameters with 95% confidence intervals using MLE and use the fluctuations in signal amplitude as the scaled MM input to a Gamma Process. A random variable *X* that is Gamma-distributed with shape *a* and scale *b* is denoted by X~Γ(a,b)≡Gamma(a,b) with the probability density function:

(2) f(x;a,b)=xa−1e−xbbaΓ(a) for x>0; a,b>0

The Gamma mean μ=a⋅b and the variance $\sigma =a\cdot b^{2}$ with the noise to signal ratio $NSR=\frac{\sigma }{\mu }=b$, which is the scale parameter that we will refer to as *the noise*.

In Figure 5D, we show the estimated Gamma family of probability distribution functions (PDF) color coded by quadrant location (whereby each PDF curve corresponds to each point in Figure 5A,B). We also mark one point as one curve and localize it according to the Kantarovich/Wasserstein distance (K/W distance) relative to a theoretical Gaussian (best) or theoretical Exponential (worst) value in Figure 5E. As noted before, these criteria were empirically determined from examining thousands of cases across multiple pathologies of the nervous system [4,7,29,42,43], also including the evolution of motor noise in neonates [41] and a comatose patient [44] to classify deliberate volition in the precise sense of physical realization of mental intent vs. spontaneous random noise.

In Figure 5E, we represent the location of the point in probability space by obtaining the distance to the empirically estimated values for the best and worst case scenario. To that end, across all body nodes and routines, we find the PDF with the highest shape value (most symmetric) and highest signal content (lowest noise) and set it as the best case scenario. We also ascertain the worst case by the PDF with the most skewed shape (closest to the Exponential) and highest noise. In Figure 5F, we can see the Gamma summary statistics obtained from the Gamma moments and color-coded according to the RLQ and LUQ regions. These forms used to visualize the stochastic features of the data can help us create objective performance outcome measures, i.e., digital biomarkers that are automatically derived from the inherent variability of the fluctuations in the movement and biophysical data.

### 2.7. Second Parameterization: Coherence-Phase-Frequency (CPF)

As mentioned in Section 3.4, the spike trains of MM reflect the normalized peaks in the order in which they were acquired. In this sense, the trajectories of some body parts may generate more curvature than those of other body parts, or may move faster or slower and generate an irregular number of speed peaks. As such, they would give rise to non-uniform numbers of peaks, such that the signal would be misaligned across different body parts (i.e., with a different number of peaks per frame, in the order in which they occurred). To maintain the originally similar number of frames (i.e., for uniformity in the computation of coherence metrics), we pad with zeros those frames with no bending peaks (i.e., where the bending is constant) or with no speed peaks (in the case of the speed). An example is shown in the right panel of Figure 4C, where the MM are plotted in the order in which they occurred, scaling all 400 peaks detected as fluctuations above the mean. In Figure 4D, these are then plotted using the original full number of frames, zero-padded where no fluctuation is detected, to produce an equal number of frames for each of the 38 body nodes of each dancer (Fluctuations below the mean can be informative in patient populations, but are more prone to be contaminated by instrumentation noise, thus requiring additional signal processing to separate the physiological signal from noise. In the bending parameter, such small fluctuations are not as informative as the large ones, but in other parameters like speed or acceleration, they tend to add other sources of information with potential classification power for pathologies of the nervous systems.) These data in the temporal domain (960 Hz divided by two dancers) are then used to convert to the frequency domain and perform power spectrum analyses.

**Figure 6:**
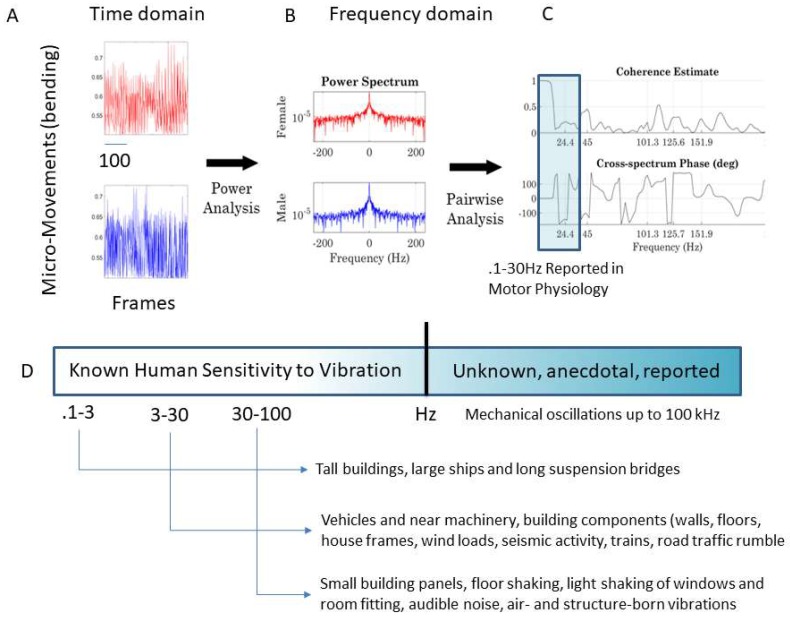
From the time to frequency domain. (**A**) The MM frames from different body nodes are FFT to perform power spectral analyses (**B**) and then pairwise cross-coherence analyses yield the frequency of the peaks (*x*-axis) and the phase shift (*y*-axis) in (**C**). (**D**) Frequency ranges studied in human motor control vs. other ranges studied in disciplines that ascertain levels of detectable vibration that may be in some cases harmful to the human nervous systems. Higher frequencies that are physiologically relevant to the vibro-tactile domain are of interest to build sensory-substitution devices.

Specifically, we conduct pair-wise cross-coherence analyses using spectral power density analyses (i.e., between any two given body parts of one dancer and any two given body parts of the two dancers). Figure 6A shows two sample MM trains for a body part of the female and one of the male. These are used across seconds with an overlapping sliding window of up to 0.5 s to perform power spectrum analyses of their FFT signal.

The number of overlapping frames is randomly chosen using the continuous Gamma family of probability distributions, generated with the MATLAB function *gamrand*, according to the empirically estimated Gamma-mean and Gamma-variance, using the MLE approach, as explained above (fitting the Gamma shape and scale parameters obtainable from the whole data set to attain the mean and variance). These continuous blocks of MM signals are used to perform pairwise cross-coherence analyses (schematics in Figure 6B,C in this case, for the pair of nodes in Figure 6A, taken from the female and male dancer).

The output of the cross-coherence in Figure 6C can then be used to construct three matrices with 76 × 76 entries, 38 for the female’s and 38 for the male’s body nodes (shown in Figure 2B). We highlight in Figure 6D the frequency range commonly examined in electrophysiology studies of motor Neuroscience and contrast the narrow frequency range examined in such studies with the broader spectrum that the human system can detect in Figure 6D, as assessed by other disciplines (e.g., structural engineering). Such disciplines ascertain frequency levels of environmental vibrations that the human nervous system is capable of detecting to shy away from potentially harmful levels of vibration in equipment, buildings, means of transportation, etc. In this paper, we examine frequency bands beyond those traditionally studied in motor electrophysiology. The motivation behind this approach is to assess other frequency ranges possibly “broadcasting” useful information from these complex dyadic behaviors that have not been previously evaluated.

**Figure 7:**
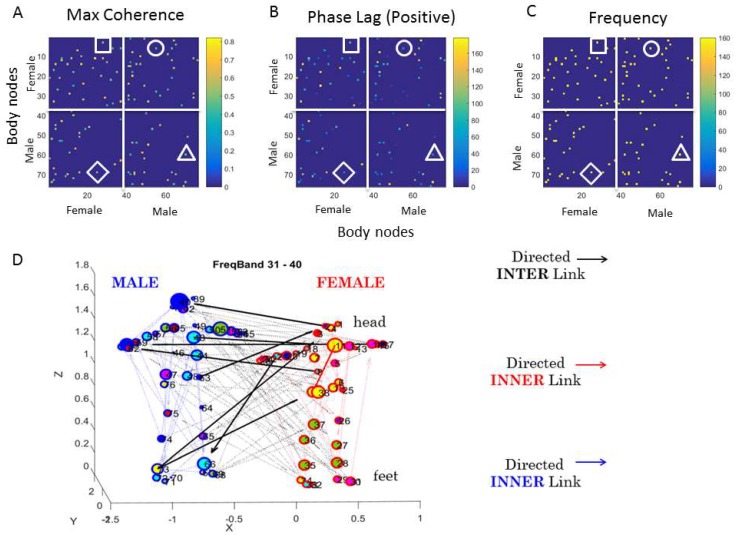
The coherence, phase, and frequency (CPF) parameterization and the weighted directed graph representation of the data as dynamically changing networks. (**A**) Adjacency matrix of 76 × 76 entries (38 for each dancer) representing the state of the two dancers in one block of MM data. Each dot represents a maximal value (a peak) of the cross-coherence, with the range of coherence values represented in the color bar. Entries with 0-values have 0 cross-coherence. Four quadrants provide the pairwise values for the female body parts (38 LEDs on the top left quadrant); for the female → male (top right quadrant); for the male → female (bottom left quadrant); and for the male body parts (bottom right quadrant.) Square, circle, diamond, and triangle in each quadrant have the corresponding values of phase lead in (**B**) and frequency in (**C**). (**D**) Network representation for a frequency band and block of MM data (see text) highlighting the interconnectivity of each body (blue weighted directed arrows male and red weighted directed arrows female). Black weighted directed arrows are the coupled activities across the dyad (thicker arrows are higher weight given by the phase lead values). Circle size is the strength of the connectivity (in degree and out degree counting number of edges entering and leaving the node) and color is the module representing highly interconnected sub-nets that are sparsely connected to other clusters.

Coherence-Phase-Frequency (CPF) Parameterization: The first matrix in Figure 7 contains, along the rows and columns, all the female nodes (1–38), followed by all the male nodes (39–76). There are four quadrants: top left represents the female-female pairwise relations, i.e., providing information about the pairwise cross-coherence of bodily biorhythms in the female. This sample sub-matrix gives information useful for discovering the female bodily synergies for each frame. For example, within this upper-left quadrant, the entry (1, 35) of the matrix given by the head (row 1) and the left foot (column 35) marked by a square, gives the value of the peak cross-coherence (0.55) shown by the color bar. These two body parts are not as coherent as those matrix entries with cross-coherence values above 0.8. Also, matrix entries at 0 have 0 cross-coherence. The corresponding (1, 35) entry in the second matrix of phase lead values of Figure 7B provides information about the phase (the head shifted ahead of the left foot 80 degrees in this case). The corresponding (1, 35) entry in the third matrix of Figure 7C provides the value of the frequency at which this occurs (in the range of 120 Hz) in this case. Clearly, the next frame will have different configurations for the female body.

The upper-right quadrant of the matrix in Figure 7A, on the other hand, provides information about the cross-coherence of the female and male body parts. There, a circle marks one example whereby entry (5, 57) (female thorax male right arm) and gives the information (as above) about the pairwise cross-coherence, phase, and frequency for a block of data comprising this one snippet of the behavior. In this way, we can dynamically sample this information across the 23 min of dancing or non-dancing conditions.

Recall here that the number of elements conforming to a block (along with the sliding window up to ½ the block size) depends on the sampling resolution of the sensors and the number of peaks we attain for the given parameter (bending or speed in this case) to empirically estimate the parameters of the underlying probability distribution function describing the block variability with tight confidence intervals. Further, the overlapping sliding window that we use is up to ½ the block size, which we obtain at random, drawing from the continuous family of Gamma probability distributions, using the estimated Gamma mean and Gamma variance from the entire data set (This use of the continuous Gamma family of probability distributions is not an arbitrary choice, but rather an empirically informed one, derived from studies involving thousands of human participants for whom we have extracted the stochastic signatures of naturally occurring behaviors at voluntary [38], involuntary [39], and autonomic [40] levels of control using linear and angular speed, linear and angular acceleration, and heart rate variability of the R-peaks.). The other quadrants offer information about the female-male pairwise coherence (lower left) and the male-male bodily coherence (lower right) for this block of data, representing a snippet of the unfolding behavior.

This parameterization of the data by coherence, phase, and frequency (CPF) offers a new way to represent the bodily behavioral data as adjacency matrices of weighted directed graphs. The directionality in this case is provided by the presence of an entry value of the second matrix under the convention that in entry (*i*, *j*), the node *i* leads the node *j*: *i* → *j*. The weight of the link between node *i* and node *j* is given by how much the phase leads, given by our convention using the positive phase shift range.

We can use a given frequency range to perform these analyses. As such, we can examine the grid of bodily nodes of the two dancers from the adjacency matrix corresponding to a given frequency range. The node here represents the amount of coherence (e.g., the edge of the circle representing a body part may be colored based on the coherence level); the size of the node may represent a quantity associated with the degree of the connectivity (incoming links, outgoing links, strength given by the net sum of incoming and outgoing links, or any connectivity-based ratio that we may build therein, etc.). The directionality is determined as explained above, by adopting the *i* → *j* convention, and the weight of the link may represent the phase shift, i.e., the amount by which the node *i* leads the node *j*, and be expressed by the thickness of the line or the type of line (dashed vs. continuous, etc.).

This network representation for one frame (block as defined above) is shown in Figure 7D based on the adjacency matrix of Figure 7A (restricted to the positive phase values of Figure 7B). This figure focuses on the frequency band of 31–40 and shows the female and male in T-pose to illustrate the configuration of the block of data we are using to present the methods. There is nothing special about this frequency band or even about the choice of connectivity parameters used to visualize the unfolding dynamics of the connectivity patterns of the network. We just chose these metrics to help explain and visualize our new methods. For example, we use the metric of modularity (sub-networks with nodes that are maximally interconnected among themselves and minimally connected with other subnets of the network as they self-emerge) to color the nodes; yet, we could have used other coloring schemas based on clustering coefficients or shortest-distance-path based metrics, etc.

Figure 7D, showing the example of the network state for a given frequency band, helps us appreciate the inner links of the male (blue colored), the inner links of the female (red colored), and the black links inter-connecting the dyad in both directions, the male and the female. These interlinks help us quantify who leads who and when (i.e., at any given block of data). Additionally, within each dancer’s body, these graphs inform which body parts of the dancer lead which other body parts. Further, the modularity analyses reveal self-emerging sub-networks of joint activity (perhaps call them inter-bodily synergies). Here, the magenta-colored module spans the left foot of the female, her left arm, and several of the male body parts (head, right shoulder, both forearms, right hip, right knee, and left shank). The arrow direction tells us which body part leads and the thickness tells us by how much.

**Figure 8:**
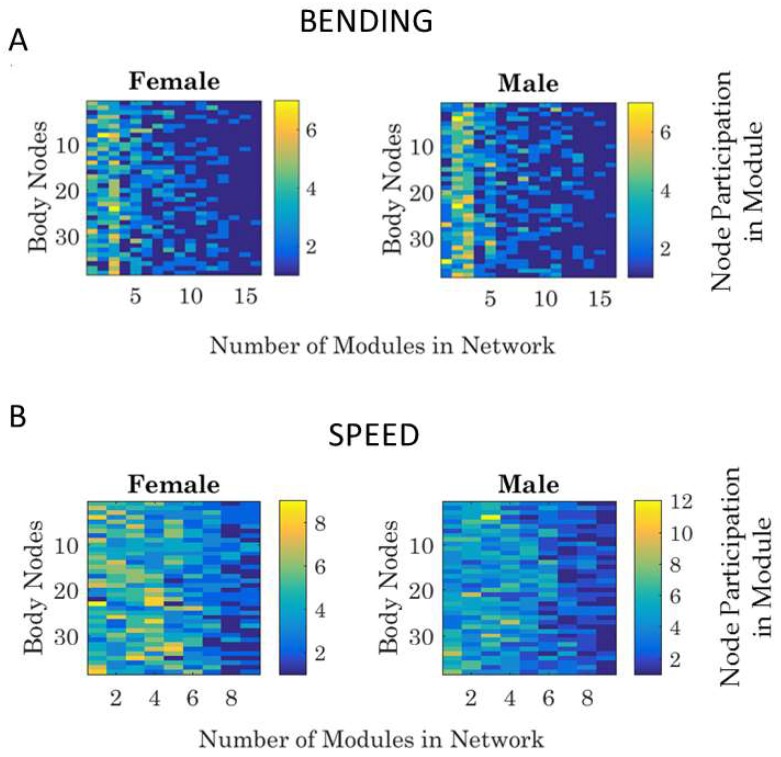
Sample use of the modularity metric across different 10-based frequency bands. (**A**) Different modules (16) self-emerge for each data block of the dynamically evolving network, as the routine unfolds for the female and male. (**B**) Counting the participation of each body node in each of the modules. The entry of the matrix (color map) gives the number of times (per units of time, e.g., minute) a node participates in a module (horizontal range from 1 to 16 from (**A**)) for the female and male dancer, e.g., during the dancing condition in this case.

We can quantify the number of modules for a network state, and within each module, we can quantify the participation of each body node in each self-emerging module. Examples are shown in Figure 8A for the bending and in Figure 8B for the linear speed networks.

Because modules may shift from block to block (i.e., as synergies would recruit and release different joints and the body would self-organize recruiting and releasing different synergies), we perform this count of node participation for each block separately. In this way, we can query how much the two bodies entrain.

### 2.8. A Measure of Physical Entrainment

To ascertain levels of physical entrainment across the two bodies in motions, we examine the space of coupled behaviors, i.e., corresponding to the inter-dancer connectivity -black links in Figure 7D. This can be done in two modes: (1) Deliberate: querying about specific body parts that synergistically interact together. This would require setting a threshold that is automatically determined by the statistical properties of the count (e.g., the median value, or the maxima, the mode, etc.) such that instances above the threshold taken across all counts are marked vs. discarding those below that threshold; (2) Spontaneous: querying via quantification of the self-emerging cohesiveness of the network’s interlinks without any pre-defined region of interest or threshold. This would merely be based on the count for nodes in both bodies and common module. In this sense, deliberately choosing regions of interest vs. letting the activity spontaneously emerge over time tells us about different types of cohesiveness, providing a flexible automated way to quantify these dyadic phenomena.

**Figure 9:**
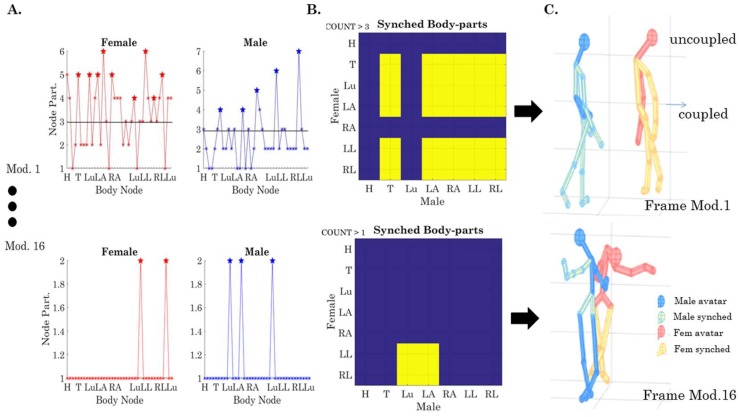
The automatic extraction of coupled synergistic behavior from the modules that self-emerge in the network’s dynamic evolution. (**A**) Simultaneous node participation in a given network module is tallied and threshold set to ½ the minimum of maximal participation. Such points count as coupled behavior for body segments that are comprised of such nodes. (**B**) Matrix representing the coupled behavior (yellow) across body regions of the two dancers. (**C**) Avatar representation of the coupled behavior. Blue (male) and red (female) with yellow segments colored from module to module. See Videos S2 and S3 showing full movies module by module and Video S4 showing the real-time dance video segment with the avatar representation.

For example, to examine (1) above, we divide the body into several regions of relevance: head (H), trunk (T) lumbar (Lu), left arm (LA), right arm (RA), left leg (LL), and right leg (RL), as in Figure 2B. Then, we can query for each module that we uncover, the node participation for the two dancers. In Figure 9, we present an example of such types of queries, enabling us to see which body regions are (maximally) participating together within the same module. Figure 9A shows the count in module 1 for each body region (comprising several LEDs, i.e., several nodes of the network). For example, to examine the thorax (T), we count the number of times that the nodes in T-male and T-female participate together by thresholding above ½ the minimum number taken across the maximal number of times/min that the nodes participate in this module (6 in the female and 7 in the male, so we have a threshold above 3). Any given body part above 3 (6/2, which is the minimum of the two maxima) falls in this set and is marked with stars as those nodes that peak together (i.e., there is a change in the sign of their slope). Then, we color code the 7 × 7 matrix (Figure 9B) by setting the entries that are above the threshold of 3 to 1 (in this module) and setting those equal or below this (self-evolving) threshold to 0. In module one of Figure 9A, we see that the trunk (T) and both arms and legs of the male (LA, RA, LL, RL) are entrained with the T and Lu and LA and both legs (LL and RL) of the female; yet, the H and Lu of the male are not entrained with any nodes of the female and the H and RA of the female are not entrained with any of the nodes of the male.

The top panel of Figure 9C presents this configuration in avatar form, whereby the coupled nodes are plotted in yellow and the uncoupled nodes are plotted in red (female) or blue (male). Likewise, module 16 in Figure 9 (bottom panel) shows the coupling of the male Lu and LA with the female LL and RL for another module in a different frame. The full movie showing the unfolding of the routine using these avatar representations of the modularity activity can be seen in Video S2 for the dancing condition and Video S3 for the non-dancing conditions. The real-time full routine can be seen in Video S4.

## 3. Results

### 3.1. Connectivity Metrics: Body-Body Networks Degree Distributions

**Figure 10:**
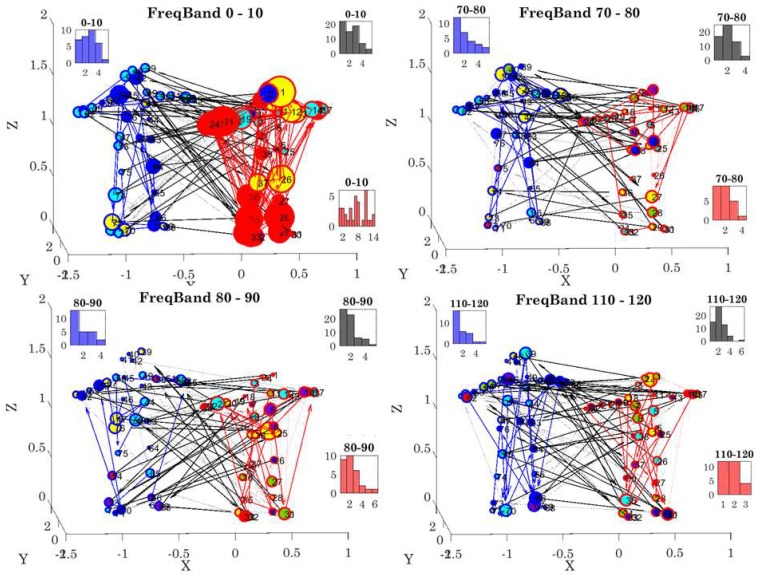
Sample degree distributions for different frequency bands in one snippet of data reflecting the state of the network differently for lower vs. higher frequencies. Each inset frequency histogram reflects the number of K edges on the *x*-axis and the number of nodes with k edges along the *y*-axis. The color of the frequency histogram corresponds to the color of the links. Red reflects the female inner links; blue the male inner links; and black the coupled network inter links. Arrows reflect the directionality and thickness the phase shift (thicker values are higher shifts). Note the differences in degree connectivity between lower and higher frequency bands for each of the subnetworks of interest.

The degree distributions of each network state were obtained by counting the number (K) of links in the network and quantifying the number of nodes with K links (edges) at each stage. We examined the 10-based Frequency range (from 0–240 Hz, 1/2 of the 480 Hz sampling rate range distributed between the two dancers at 960 Hz) for 23 cases. Figure 10 shows four different cases (for simplicity), whereby the associated degree distributions (shown as insets) for the female (red), male (blue), and coupled (black) networks are shown as frequency histograms of the number of K edges in the network (horizontal axis) and the number of nodes with K edges (vertical axis). Note the changes in these distributions across different frequency bands. Additionally, note the changes in modularity, directionality, phase, and coherence values for each frequency band.

**Figure 11:**
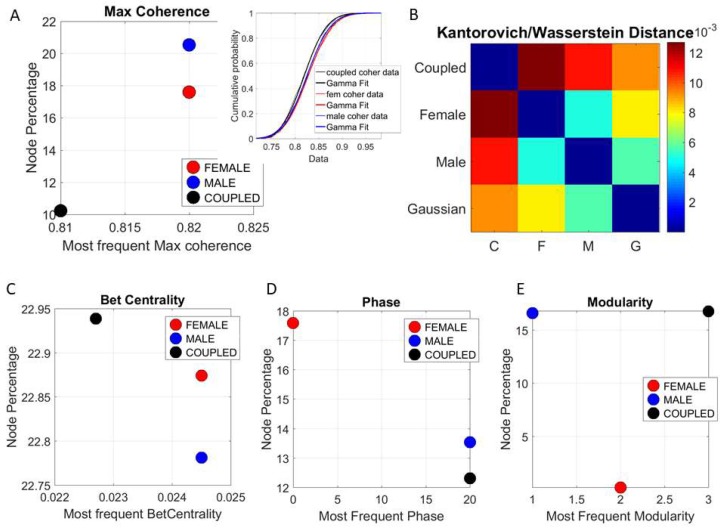
Connectivity metrics measuring female, male, and coupled networks: (**A**) Percentage of nodes with maximal coherence for the coupled network significantly differed from the networks of the male and female bodies; (**B**) Pairwise K/W distance matrix reveals patterns for the female network, the male network, and the coupled network, relative to the ideal Gaussian signature; (**C**) Patterns of betweenness centrality (**D**) phase and (**E**) modularity are also different for each network under consideration. Notice the female network the has highest percentage of nodes with 0 phase shift (fully synchronous) and the coupled network has the highest percentage of nodes with the highest number of modules.

These results prompted us to track the patterns of coherence, phase, and connectivity metrics (e.g., betweenness centrality and modularity) for each of the subnetworks of interest (female, male, and coupled) shown in Figure 11. We quantified the distribution of coherence peaks across the network nodes across all states of the network. We found statistically significant differences for the percentage of nodes with maximal coherence (non-parametric rank sum test *p* < 0.001) for the coupled network and the male or female networks. This is also appreciated in the inset profiling the empirically estimated cumulative distribution functions of each network. The Kolmogorov-Smirnov test confirmed statistically significant differences between the coupled network (*p* < 0.001) and for the female or male networks, with regards to the % nodes with maximal coherence. A higher percentage of maximal coherence was found across the nodes of the male network but was not significantly different from the female one, unlike the coupled network, with a significantly lower % of nodes with maximal coherence.

Further, we examined the K/W distance for the probability distribution functions best fitting the frequency histogram of the nodes’ maximal coherence. The results are shown in matrix form in Figure 11B with respect to the theoretical Gaussian distribution. The closest to the Gaussian case was the male network, while the coupled network was the farthest. Figure 11C–E shows the node percentage for betweenness centrality, phase values, and the modularity metric for the female, male, and coupled networks. There, the coupled network had the largest number of modules and the highest node percentage participation in the modules.

### 3.2. Connectivity Metrics: Body-Body Networks Leading-Lagging Profiles

We examined the degree to which each body part of interest (shown in Figure 2B) was leading for a given frequency band and which dancer was leading in general for each condition. To that end, the number of links leaving the node (out-degree) was obtained, considering the weight of the edge (the phase shift), and the leading profile was denoted. This weighted sum accumulated over a session (per each frequency band) provided a metric of the strength of the body part leading another body part (where all nodes point in one direction according to our *i* → *j* convention).

**Figure 12:**
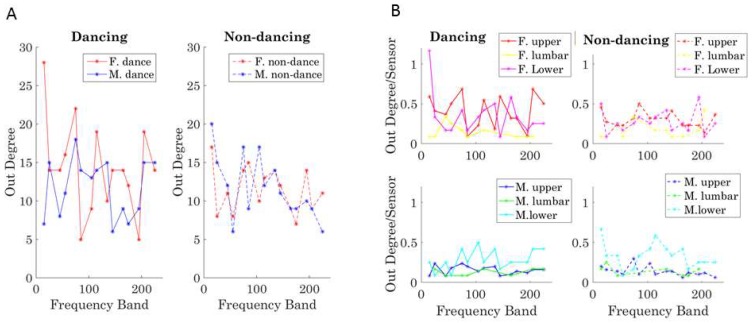
Automatically detecting patterns of leadership in the cohesiveness of the coupled behavior (inter-connectivity in (**A**) and intra-connectivity for each dancer in (**B**). (**A**) Leading patterns across the full network of spontaneously self-emerging coupled nodes across 10-based frequency bands. Patterns unveiled using the outdegree distributions and including cohesive activity in inter-connected nodes across the two dancers and (**B**) Leading activity for the self-emerging synergies of the body of each dancer (intra-connectivity). Out-Degree per node (sensor) used to unveil the leading information for selected body regions (upper body including the head, trunk, arms and hands), lumbar region (including lumbar areas and the hips) and lower body region (including the legs and feet) of each dancer. For the dancing condition in (**A**) the female tends to lead across frequencies with the male taking the lead for 90–100 Hz range. In contrast the non-dancing condition is primarily led by the male dancer, except in bands 90–100 Hz and 190–210 Hz. In (**B**) the individual patterns reveal which bodily region leads within each dancer’s synergies and condition, per frequency band.

The leading profile can thus be studied for each dancer across the dancing and non-dancing conditions by taking the overall sum. We plot the summary for each frequency band in Figure 12A for the dancing condition (left) and the non-dancing condition (right). Further, the break down per body segment (upper body, core denoting lumbar and trunk, and lower body) is shown in normalized form in Figure 12B, where the dancing (left) and non-dancing (right) conditions are shown separately for the female (top) and the male (bottom). This normalized version expands on the results in Figure 12A, where the periodic patterns of this measure show the female leading in the dancing condition for most frequency bands and the male body leading in the non-dancing condition. Figure 12B informs us of the male and the female interactions according to body segments. For example, the female lower body leads in the lower frequency band below 10 Hz, but then the upper body leads in higher frequencies for the dancing condition. The lower body of the male, on the other hand, leads for most frequency bands in both the dancing and non-dancing cases. This metric can be further refined by body parts to provide other profiles of the weighted-directed networks’ lead-lagging patterns.

### 3.3. Dynamically Coupled Body-Body Networks

The analyses concerning the togetherness metric in Figure 9 provide a frame-by-frame characterization of the dancers’ entrainment and serves to aid visualization of a very complex routine into simpler modules, as these modules dynamically emerge and dissolve across the routines. Here, we underscore that the patterns emerged automatically for body parts we searched for, as the dancing or no-dancing conditions unfolded. If the end-user (e.g., researcher or choreographer) would like feedback on other body parts, it is also possible to redefine the areas of interest across both bodies and profile those instead. In this sense, the method allows ample flexibility without sacrificing the automated aspects of self-emergent cohesiveness captured in the modularity metric of the network. Figure 9, used to explain these methods, shows this metric for a couple of selected frames. For the complete movies, please see Videos S2 and S3.

### 3.4. Automatic Identification of Connectivity and Coordination Patterns

Across the different 10-based frequency bands, we computed the shortest distance paths (between nodes) as the network dynamically unfolded during the dancing condition. The distance matrix obtained using the connectivity toolbox of MATLAB [45] contains the shortest paths between all pairs of (*i*, *j*) nodes in its (*i*, *j*) entries. This information was used to estimate the profile of the characteristic pathlength (the averaged shortest distance paths across the network) and examine this value per frequency band.

**Figure 13:**
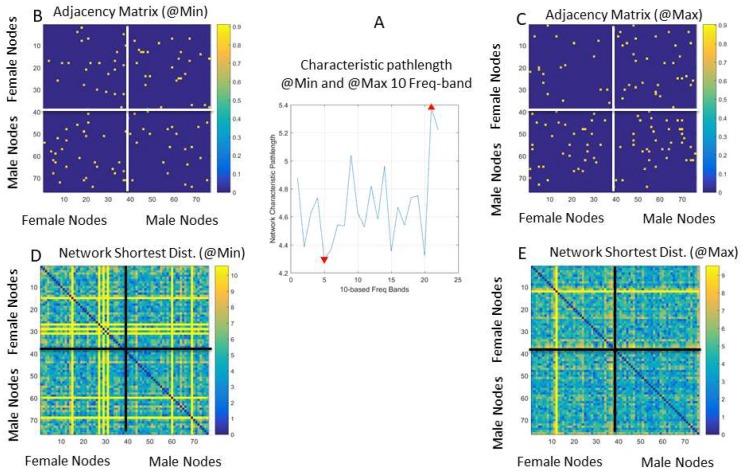
Network’s self-emerging connectivity patterns for different 10-based frequency bands uncover critical network states. (**A**) The characteristic pathlength of the network measuring the average shortest distance path profiled for different frequency bands. (**B**) Adjacency matrix denoting the weighted directed graph used to build the network states for the minimum and maximum characteristic pathlength revealing the frequency band for which they occur. (**C**) The network pairwise shortest distance paths when the characteristic pathlength is at its minimum value (which happens to occur at 50 Hz). (**D**) The network’s pairwise shortest-distance-path state when the characteristic pathlength is at its minimum value (occurring at 50 Hz). (**E**) The network’s pairwise shortest-distance-path state when the characteristic pathlength is at its maximum value (occurring at 210 Hz).

In Figure 13A, we can see the profile of the network characteristic pathlength across the 10-based frequency bands. The network characteristic pathlength is the average shortest distance path. This value changes periodically across the 23 10-based frequency bands that we explored. Here, the minimal value and the maximal values denoting critical transition points between frequency bands reveal different states of the network captured by the adjacency matrices from the weighted directed graphs in Figure 13B,C for the minimal and maximal characteristic path length of the network, respectively. There are many other connectivity metrics that we can examine using this schema to automatically reveal frequency bands that can transmit information about connectivity patterns.

Here, in Figure 13B,C, the dots are the values of the maximal coherence and underlying these values are weights denoting the positive phase shift of node *i* over node *j*, which we use by convention to denote leading directionality, i.e., that the body part represented by that node *i* leads the body part represented by that node *j*. As such, the maximal coherence at entry (*i*, *j*) with the shortest weighted edge path is also maximally synchronous and *automatically* reveals patterns of synergies across the dancers and/or within the body of each dancer, without any heuristics (e.g., having to choose a specific number of wavelets, or thresholding values in phase locking-based methods, etc.).

The network’s state corresponding to the shortest distance path can be appreciated for the case of the minimal characteristic pathlength (occurring at 50 Hz) in Figure 13D. There, the female network is on the top left panel; the male network is on the bottom right panel; and the cases of female → male and male → female are on the top right and bottom left panels, respectively. The network’s state at maximal network characteristic pathlength occurred at 210 Hz in Figure 13E. These analyses reveal different coordination patterns for each dancer and across the dancers’ coupled network for different frequency bands (within the 240 Hz range under examination). Such different frequency bands can then be used for real-time transmission without interference, confusion, or cross-talk between the frequency channels, given how different the connectivity metrics are at these extrema.

**Figure 14:**
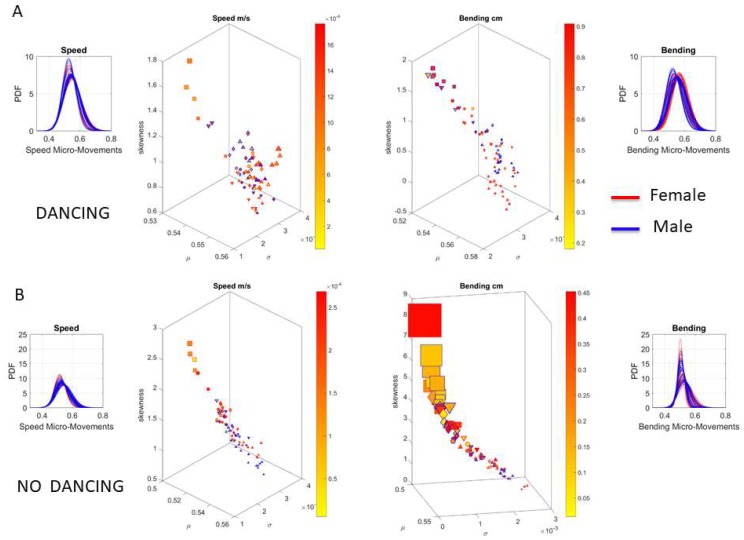
Summary statistics for the empirically estimated continuous family of Gamma probability distributions for speed and bending profiles taken for each of the 76 body nodes for the female and for the male dancer, by pooling movement activity across the 14 dancing routines (**A**) and non-dancing (calibration and planning) segments (**B**). Color bars represent physical levels of fluctuations in linear speed (cm/s) and bending (cm) measured as departures from the overall estimated Gamma means. Axes are as explained in methods (*x*-axis Gamma mean, *y*-axis Gamma variance, *z*-axis skewness and kurtosis represented in the size of the marker; squares represent lower body, triangles upper body, and circles head LEDs; blue edges represent male and red edges female).

Underlying the network connectivity activity of each contributing node are patterns of the signal to noise ratio (mean to variance ratio empirically estimated from the Gamma process.) We plot the Gamma statistics summary from the empirically estimated Gamma moments in Figure 14A for the dancing condition (i.e., pooling movements across all routines), with the linear speed patterns on the right and the bending patterns on the left. The corresponding PDFs are also represented. Furthermore, Figure 14B shows the patterns for the non-dancing condition with a higher spread in the skewness and kurtosis values.

### 3.5. Individualized Noise-Body-Map Profiles

The node’s activity underpinning the network connectivity patterns also contributes to our understanding of the dancer’s coordination patterns and the statistical quantification of the emerging families of probability distributions that we empirically estimate. The evolution of the noise to signal ratio across frames can also be captured and mapped throughout the dancer’s performance.

**Figure 15:**
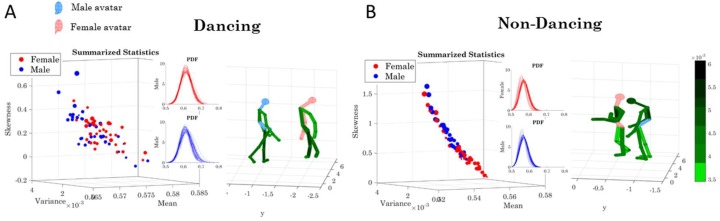
Bodily maps of noise (scale values) for the female and male using the bending profiles pooled across all routines for each body node. (**A**) Dancing condition with insets showing estimated PDFs for female and male. Avatars color-coded for one frame with levels of noise for the physically entrained body parts as explained in Figure 8 (blue avatar male, red avatar female, and green color gradient as in color bar in (**B**), also showing the non-dancing condition).

In addition to the pooled data across routines, we can also selectively map the noise profiles across the body nodes and use an avatar-body representation to visualize these patterns frame by frame. Figure 15A shows this representation for a frame of the dancing condition, while Figure 15B shows it for one frame of the non-dancing condition. Full movies can be seen in Videos S5 and S6. Table 1 shows the statistically significant differences between the empirically estimated shape and scale parameters of the Gamma distribution for these two conditions.

### 3.6. K/W Distance in Probability Space

**Figure 16:**
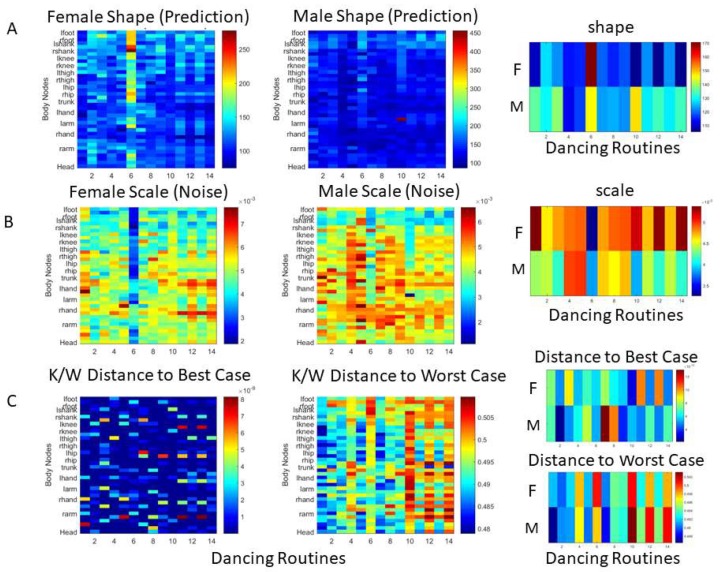
Dancing routine performances at a glance, measuring the K/W distance from each dancer’s empirically estimated probability frequency distribution to the best or to the worst empirically determined PDF, as ascertained by noise levels and distribution shape symmetry across the entire set. (**A**) Profile of empirically estimated Gamma distribution shape from female dancer’s linear speed across 14 routines (horizontal axis) and 38 body nodes (vertical axis) readily reveals routine 6 as the one with the most symmetric distribution. Male profile as female’s. Averaged Gamma distribution shape values taken across all body nodes for each dancer confirm routine 6 as the one yielding the most symmetric distribution shape. (**B**) Same as (**A**) for the Gamma scale parameter, empirically estimated from the linear speed profiles of each routine and across the grid of body nodes. (**C**) K/W distance from routine and body node to the best (and worst) values taken across entire data set as the highest shape (closest to Gaussian symmetric shape) and lowest scale (noise) used to define the best case vs. the most skewed shape (closest to Exponential) and highest noise used to define the worst case.

The results of computing the maps of shape and scale for each of the 14 dancing routines are shown in Figure 16A for the female (first) and male (second panel), with an averaged summary displayed in the third panel. Figure 16B shows the representation of the scale (noise) across all 14 dancing routines for the female, male, and averaged summary across all 38 body nodes. In both cases, we can see that routine 6 emerges as the highest shape value (the most symmetric) and the lowest scale value (the least noise), with a detailed representation of the K/W distances in Figure 16C. This figure shows the K/W distance to the best case scenario (towards the Gaussian limiting case to the right) and worst case scenario (towards Exponential limiting case to the left), as unveiled by the data (see methods for criteria). This figure confirms that across body nodes, routine 6 was close to the best case scenario, but also routines 10, 12, and 14 resulted in patterns with a close distance to the best case, and were consistently far from the worst case scenario. Within the quantification of distance in probability space, we can also see which specific body nodes are closer to, or farther away from, the critical points detected across the full set of routines. We underscore that these notions of similarity or difference self-emerge from the stochastic signatures of the inherent fluctuations of the movement data. As such, they provide automated ways to examine, in these complex dyadic interactions, which routines give rise to the highest signal content with maximal predictability: a form of kinesthetic feedback that we can monitor and correct in real-time.

## 4. Discussion

This paper integrates a new statistical platform with new data parameterizations and new visualization tools developed for research requiring real-time updating of biophysical signals with inherent stochastic shifts. The methods are generalizable to multi-modal sensors co-registering activities from various levels of the nervous systems. Besides the peripheral bodily signals studied here in coupled behaviors, they can also handle biophysical signals from the autonomic nervous systems (e.g., heart rate variability [46]) and the central signals from electroencephalography (EEG) [46,47]. Here, we used wearable active LEDs on body suits detectable by high-speed cameras sampling at 960 Hz. However, accelerometer data from inertial measurement units (IMUs) using grids of wearable sensors sampling synchronously have also been used to test these methods in autism diagnosis, see, e.g., [25,46,47] and Appendix A and closed-loop interfaces involving partnering dancers in pop music [48] (Appendix B).

These analyses informed us in a fast and simplified manner about the automatically entrained or de-entrained body parts in each dancer, for different frequency bands. Most importantly, they revealed the dynamically evolving coupled cohesiveness of the two bodies in motion, a type of information invisible to the naked eye of a person coding videos, or of an observer describing behavioral exchange (social or otherwise) between two people (or two animals in an animal model of behavior). Video-coding or video-annotation is commonly used in contemporary ballet, where imitation is one of the means to teach choreographies across different schools. There is no standard vocabulary or grammar of annotations as they are specific to each school. Furthermore, former methods such as Laban notation [49] or Benesh movement notation [50] are rarely used today owing to a lack of general consensus for their use, and a variety of existing styles from school to school. The decline in the use of prior annotation styles combined with the emergence of new sensors amenable to tracking complex motions unobtrusively provides a good opportunity to introduce the standardized platform that we provide here to the ballet community. Our real-time methods permit the direct layout of a video, with avatars representing synergies and coupled behaviors (Video S4), and offer new scales suitable for creating a standard vocabulary of basic motions to build and teach choreographies, and to track the performance level of one dancer with respect to another dancer, or in relation to a full ensemble.

The present approach to dyadic interactions fundamentally differs from traditional methods. Unlike traditional methods that adopt a “one size fits all” model to analyze statistical effects of a given treatment, e.g., to ascertain their statistical significance, the present approach is personalized. The new methods empirically estimate, for each individual, the families of probability distributions that from moment to moment, best fit the fluctuations of the bio signals self-generated and self-monitored by that individual’s nervous systems. Unlike current methods assuming a priori a theoretical probability distribution to summarize the statistical properties of the coherence phenomena, or pre-setting a fixed number of wavelets to carry on the analyses, here, we empirically determine the families of PDFs as the behavior unfolds in real-time, without heuristics. The heuristics of the methods are based on optimization criteria derived from the data’s inherent statistical variations (e.g., criteria to stop gradient descent in MLE or initial seed for optimization are derived from the data itself). We assess the shifts of the biophysical signals’ signatures in parameter spaces using appropriate distance metrics. This approach to the motor control problem more generally enables us to adopt fully automated closed-loop interventions to individually adapt treatments to each person, based on the inherent variability of the person’s nervous systems, free of pre-set heuristics or parametric models.

### 4.1. Connecting Central and Peripheral Signals of the Nervous Systems

The methods extend the use of binary undirected graphs for network connectivity analyses commonly used in brain research (imaging, EEG, etc.) to weighted directed graphs, enabling analyses of complex full-body movements from one person and/or from coupled dyads. In this sense, we provide the quantification of leading profiles based on weighted-directed network evolution and the nodes’ underlying stochastic profiles. The dynamic nature of these data is amenable to providing movies of: (1) the network performance (see Videos S2–S4); (2) the automatically identified togetherness that the end-user can query across body segments of interest (see Videos S2 and S3); and (3) the stochastic evolution of the NSR distributed across the grid of nodes of both dancers (e.g., Figure 15 and Videos S5 and S6). These movies can provide the temporal real-time evolution of the personalized inner-networks’ synergies of each dancer; or of the inter-networks’ synergies in coupled mode, i.e., from the coupled network modularity analyses of the inter-links across the dancers (black links in Figure 7 and Figure 10).

The dynamically changing coordination patterns of inner-body and inter-body synergistic coupling were automatically extracted from the inherent variability in movement trajectory parameters. Here, we used the linear speed and the bending profiles extracted from the curvature of the positional trajectories. These were our time-series of parameters of interest, but other kinematic parameters (e.g., angular speed, linear and angular acceleration, etc.) could also be used, as they too give rise to time-series of peaks and valleys amenable to being represented as a Gamma process. Furthermore, other waveforms from EEG or ECG, etc. could be converted to MM spikes and used within the weighted directed graphs framework (see e.g., [25,47,51]). Because their use is completely automated, the methods require no thresholding, a priori determination of a specific number of wavelets, or other pre-set heuristics that other coherence analyses may employ. The coupling behavior spontaneously self-emerges from the cohesiveness of the networks representing the dyad. These tools may pave the way to studying non-linear complex coupled dynamics in a broader sense, including patterns of coupled interactions in social bodies, such as those of a theater involving the audience and the performers; or patterns generated by social crowds in naturalistic settings.

### 4.2. Other Applications in AI and Robotics

The type of empirical data one could gather using the present approach may inform models of artificial agents (e.g., avatars and robots) about properly parameterized and personalized spontaneously self-emergent behaviors conducive of autonomy/agency of the brain over one’s own body in motion, or over the moving bodies of others, when leading/lagging in a social exchange. Indeed, leading/lagging profiles are automatically extracted with the weighted directed network approach that we introduce here as a new tool extending previous work (e.g., involving central signals represented as brain networks) to peripheral signals read out from the body in motion. Such analyses re-interpret brain-body loops as closed-feedback-loops where kinesthetic re-afferent feedback is continuously updated in real-time from bodies in motion. Under this treatment of the problem, our methods were able to distinguish between the well-rehearsed dancing and the spontaneously improvised non-dancing conditions (Table 1, see also Appendix B with other dancers and salsa-dancing). In the case of ballet data, this distinction was possible, despite the fact that both dancing and non-dancing conditions are composed of deliberate and consequential motion segments. Indeed, the stochastic signatures of rehearsed routines were different from those embedded in spontaneous overtures between the dancers, during the intermezzo/calibration times.

This type of peripheral activity can be conceptualized as reafferent visual, auditory (vibrations at frequencies we can make audible or sensible through touch channels), and kinesthetic feedback of coordinated bodies in motion. The use of these new methods could be easily adapted to clinical settings, to capture the interactions between the clinician and the patient (see Appendix A real-time tracking of a type of autism diagnosis and [25]). They could also be used in medical education settings, to quantify social rapport and motor reciprocity during patient-doctor interactions, whereby the doctor can receive feedback on the patient’s physical engagement in the conversation, via the physical biorhythms of the motor output from both participating agents of the dyad. Other dyadic settings such as those in the classroom (teacher and pupil), basic research settings (researcher and subject), and home environments (caregiver and patient) could be used. Because of their automated feature and ease of computation, they are at present used in the context of personalized smart health (e.g., using smart shoes) that employ cloud-based updating [52].

These methods can also be extended to animal models whereby the interactions across animals in a colony could be monitored using camera-based systems (as those used here) or wearable sensors (as in [25,47].) Indeed, the dyadic exchange is ubiquitous to many settings and can serve as a basic unit of other more complex non-linear dynamical systems. Because we explore these biometrics at a macro-level of behaviors and also at the stochastic micro-level of spikes’ variability underpinning these behavioral patterns, we offer a new unifying framework to connect multiple layers of complexity, from cortical spikes to bodily/heart spikes, through a common analytical and visualization platform, e.g., amenable to use with grids of multimodal wearable biosensors.

### 4.3. Closing the Feedback Loop: Shifting from Correlation to Causation in Statistical Inference

The MM spikes were inspired by the predictive properties of cortical spikes during planning activity before movement onset [53], thus suggesting a good data type and framework for forecasting the sensory consequences of intentional movements embedded in the nervous systems’ self-generated activities. As mentioned, this new data type provides new ways to connect peripheral signals with cortical signals analyzed under a common statistical platform (e.g., under the general rubric of Poisson random processes, or other types of stochastic processes). This is important because most of the neuroscience work involving cortical spikes and kinematics is analyzed separately and then correlation analyses between averaged spike rates and averaged values of motion parameters ensue. By using a common statistical platform and similar data type (time series of MM spike times [54] or MM spike amplitude [7]) across multiple layers of the nervous systems, and by adopting the von Holst principle of reafference to guide our work, we can adopt the machinery of computational neuroscience and add forecasting and quick detection methods [55] to better predict the potential sensory consequences of impending actions. Such methods permit us to better infer and interpret impending sensory activity of the bodies in motion within a closed-loop setting. This setting under the unifying framework that we offer here affords causal inference of intended bodily motions from consequential spiking activity, beyond mere correlational statements of the more traditional open-loop approaches.

Most research concerning dyadic exchange is performed in open-loop mode, i.e., using tools that describe statistical phenomena a posteriori, in a rather “photographic-like” static way. Such an approach does not seem to be compatible with the “movie-like” dynamic nature of the coupled activities. The present methods allow us to go beyond a content description of correlations, and tap into real-time tracking and interventions to drive the system in parametric mode. By not imposing a priori the statistical parameters of theoretical probability distributions under stationary assumptions and assumptions of homoscedasticity, we open a new type of behavioral analysis that allows the tracking of biophysical signals’ stochastic shifts in personalized mode. Instead of throwing away as noise important fluctuations in the signal (via smoothing through grand averages, using a theoretical population mean), we can now tailor treatments to the person’s best capabilities. As such, we open a new way to reshape social interactions in real-time. This is a much needed framework in neurodevelopmental disorders, e.g., those on a spectrum that eventually result in problems with social interactions.

Furthermore, we used an appropriate distance metric to measure the changes in probability density functions represented as points on the probability function’s parameter plane—i.e., the space of transitions reflecting shifts of non-symmetric probability families empirically characterizing the motion signal. As such, by following the path of maximal change, bringing the signals closer to proper neurotypical probability regimes, this new method enables optimization approaches to select the best form of sensory guidance that will most likely drive the intended motions to accelerated positive gains in a treatment’s outcomes [3,6,48]. In the case of the partnering ballet example discussed here, we can automatically select the best routine that guides the coupled behavior towards the minimum noise-to-signal profile and the maximal tendency away from the memoryless Exponential and noisy regimes (which prior work involving autism identified as the worst case motor noise scenario [7,26] and athletes provided criteria for how to move away from it, towards Gaussian regimes [28]). In the case of the ADOS dyadic performance in the Appendix A example, we can automatically select the ADOS tasks of a given module that improves the social exchange towards one, whereby the autistic child takes the lead and produces motions with predictive signals. In this sense, the present framework opens a new way to extract in near real-time from the motor stream, the type of kinesthetic sensory guidance, routine, context, or activity that most likely tends to shift the statistical signatures of an intended and/or of a consequential motion towards appropriate neurotypical regimes. This offers new personalized means to selectively tailor dyadic-based interventions to the nervous systems’ preferences and predispositions [6,48,56,57].

### 4.4. Higher Frequencies and Their Possible Uses in Sensory-Substitution Interventions

The detection of coupled signals at higher frequencies (e.g., Figure 10) enables the parameterization and re-parameterization of these signals across sensory modalities. For example, we can move from the kinesthetic-movement domain to the kinesthetic-auditory domain via sonification of the real-time motion and blending of the sonified motion with music [48], while tracking and purposely steering the stochastic signatures of the biorhythms towards a targeted regime. This is amenable to sensory substitution and sensory augmentation methods.

We can also endow avatars with shifted versions of the real-time motions and provide re-parameterized visual feedback through various media [3,6], all driven by the real-time stochastic parameters of the motor signals to explore the person’s preferences. Using the present methods, we can transform the parameterized motor output into vibrations within selective frequency bands, and supply it to the system as sensory-feedback to help dampen the motor noise according to personalized noise profiles in relation to desirable noise regimes. These aspects of the application of the real-time closed-loop reparameterization of the biophysical signals are out of the scope of the present paper, yet they are a part of our currently active funded research program.

The advent of wearable biosensors and off-the-shelf camera systems are among the many tools offering biophysical signals from which MM spikes and CPF parameterizations can be derived. In future studies, other pipelines of analyses have the potential to unveil physiologically relevant frequencies using, e.g., subspace methods [58] that overcome limitations of the FFT-based methods. Further, time domain analyses may also complement this work and help detect similarities in the patterns of the time-series to forecast their stochastic regimes. The important point is that we have initiated new ways to standardize and scale the bio signals across different classes of wearable bio sensors and extended the use of networks and connectivity analyses from the brain [59] to the full body in motion [42]. As such, we have now developed a new way to visualize central-peripheral coupled activity and dynamically detect and track self-emergent coordination patterns between two (or more) bodies in motion. We have done so as these bodies in motion perform natural activities *in real-time* without any a priori enforced assumption on the nature of the random processes governing the nervous systems’ continuously updated signals.

These new methods and paradigms may serve to unify and advance more than one field and area of enquiry in basic neuroscience, the performing arts, the clinical areas, and the education sector. We hope that our new tools can be used to explore interacting bodies in motion in general. These may include gaming, VR, humans in social contexts, and hybrid interactive-co-adaptive interfaces of human-avatar and human-robot environments.

## 5. Patents

Torres (2016) Connecting peripheral and central nerves output signatures of variability through the same statistical platform. US patent application 62/409,943 filed 10/19/2016 (International PCT/US17/57365 field 10/19/2017).

## Figures and Tables

**Table 1:** Statistical Comparison of Dancing vs. Non-Dancing Conditions (Rank Sum Test).

Parameter	Condition	*p*-Value
Shape (*a*)	Dancing vs. NonDancing	1.3027 × 10^−21^
Scale (*b*)	Dancing vs. NonDancing	2.4329 × 10^−^^18^

**Figure A1:**
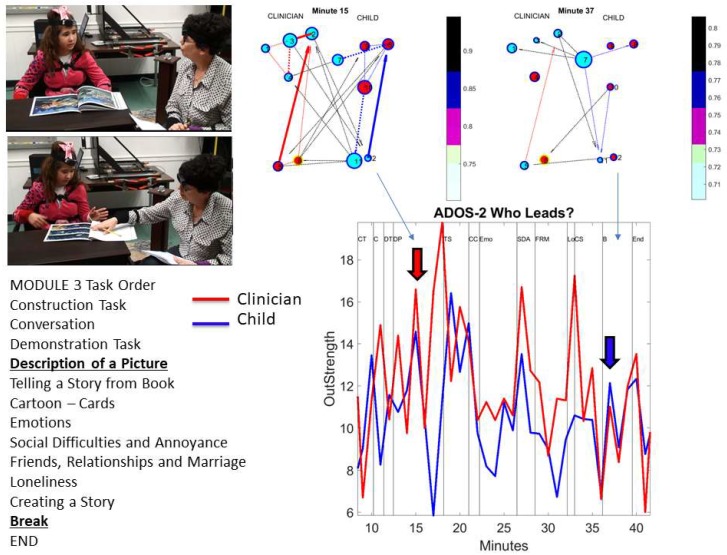
Automatically tracking who leads in the dyadic interaction taking place as the ADOS-2 test [60] unfolds (to diagnose autism spectrum disorders). Two frames depicting the clinician and child engaged in the test. Both participants are wearing a grid of APDM Opal IMUs 128 Hz, Portland OR, on the torso, lumbar, right and left wrists, and right and left ankles (along with positional head and wrist Polhemus Latus sensors, 188 Hz, Colchester VT). The 12 IMUs simultaneously co-register acceleration, gyroscope, magnetometer, and temperature data. Acceleration data is used here to demonstrate the minute-by-minute states of the network conformed by the 12 nodes (1–6 on the clinician and 7–12 on the child). The ADOS-2 Module 3 for the verbal child was administered with tasks in the order depicted and using the task initials to represent the minute-by-minute activity. The network outgoing strength (obtained from the weighted sum of the links outgoing from a node *i*, times the maximal coherence value to another node *j*) defines a profile revealing leading activity for the child and clinician. For simplicity, the network states for the corresponding minute-peak activity are shown. One is for the peak at minute 15—when the clinician leads the child (during the Description of a Picture, DP task) and the other is at minute 37—when the child leads the clinician during the Break, B task. Vertical lines delineate the tasks’ segments (denoted by the initials) in the order in which they occurred. The labeling and analyses are completely automated.

**Figure A2:**
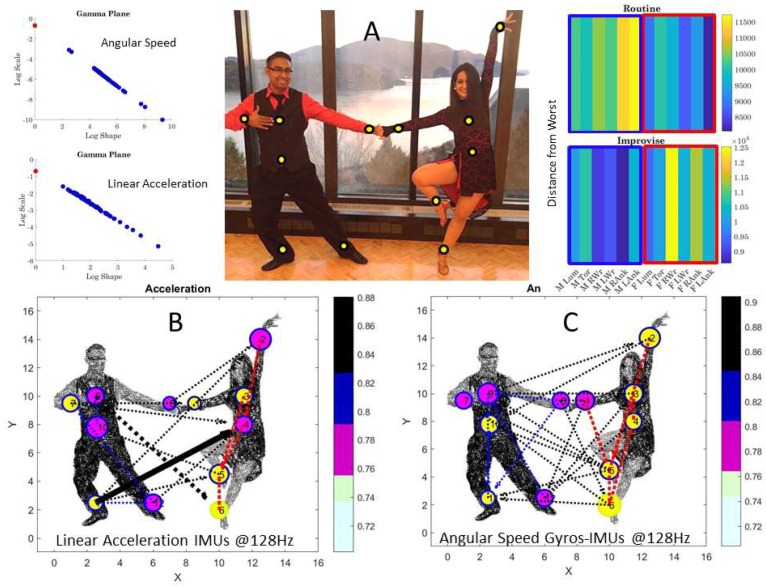
Network connectivity analyses of salsa dancing using a grid of IMUs of a commercially available sampling resolution. (**A**) Dancers in pose with sensors’ placement. (**B**) Network state derived from 30 s worth of data (30 × 60 × 128 frames at 128 Hz sampling resolution) with similar representation as Figure 7 in main paper. MM spikes derived from the fluctuations in linear acceleration amplitude. Linear acceleration time-series obtained from triaxial acceleration using the Euclidean norm to compute the scalar acceleration amplitude. Node at locations represent two self-emerging modules (colored in magenta an d yellow). Circle size represents the strength of the connectivity (incoming and outgoing weighted directed links from the node). Circle edge represents the maximal cross-coherence value measured pairwise with the connecting node. Pairwise links between nodes represent node *i* leading node *j* (*i* → *j*) and thickness represents the phase shift (lower values are thinner dashed lines 0–60, 61–120, and higher values 121–180 are thicker continuous lines). Link colors represent inner connections (red female, blue male) and black links represent inter connections from coupled nodes. (**C**) Similar representation as in (**B**) using orientation data. Data comes from the angular speed derived from quaternions representing the rotations of gyroscopes. Insets above B localize the signatures on the Gamma parameter plane relative to the worst case scenario of exponential distribution shape = 1 with the highest possible noise (extracted from the session) for angular speed and linear acceleration. Corresponding distances from each point to the extreme (most random and noisiest) case are expressed as color-coded vectors above panel (**C**). Notice the complementary patterns between the well-rehearsed routine (male body leads and has strongest predictive signal vs. spontaneous improvisation whereby female leads with a stronger predictive signal than male).

## Supplementary Materials

The following are available online at http://www.mdpi.com/1424-8220/18/9/3117/s1.
