# Supplementary material for: Peripheral Network Connectivity Analyses for the Real-Time Tracking of Coupled Bodies in Motion
Source: Sensors (Basel). 2018 Sep 15;18(9):3117. doi: 10.3390/s18093117 (PMC6164645; doi:10.3390/s18093117)
Supplement: Supplementary file 1 [file sensors-18-03117-s001.pdf]

Supplementary Materials:

The following are available online at

(1) Dancers during T-pose and calibration Video S1

https://www.youtube.com/watch?v=llyVVT_1x_4

(2) Modules and coupling behavior with avatar representation for Dancing condition Video S2

https://www.youtube.com/watch?v=XbPbUmu8ZZ0

(3) Modules and coupling behavior with avatar representation for Non-Dancing condition Video S3

https://www.youtube.com/watch?v=MJw_UHwMWBk

(4) Dancers and avatar during a routine Video S4

https://www.youtube.com/watch?v=pHJ8qYgCg0k

(5) Avatars' representation of the NSR bodily maps Dancing condition Video S5

https://www.youtube.com/watch?v=FY98PxsDIdQ

(6) Avatars' representation of the NSR bodily maps Non-Dancing condition Video S6

https://www.youtube.com/watch?v=2qXg8bRQZ8c

Table S1: Segments of raw data (some had to be cleaned of occlusions)

**Name**	**Dance segment**		**Length (Frames)**	**Length (sec)**	**Link**
	First Frame	Last Frame			
**Routine1**	16000	19300	6710	13.33	https://youtu.be/KHp5uyawm4Y
**Routine3**	12890	19600	6710	13.96	https://youtu.be/_zbKB_vYOTc
**Routine4**	6600	10800	4200	8.75	https://youtu.be/pK8tTc76lr4
**Routine5**	5500	17900	12400	25.83	https://youtu.be/73DsonVp5BU
**Routine6**	8000	48400	40400	84.16	https://youtu.be/yC0nTQBrfQ4
**Routine7**	7400	22800	15400	32.08	https://youtu.be/wNYZrzy26yU
**Routine8**	25000	52500	27500	57.29	https://youtu.be/yKREUOi_mWs
**Routine9**	5200	10500	5300	11.04	https://youtu.be/9RVJQYO4nWE
**Routine11**	1200	19500	7500	15.62	https://youtu.be/b8kFFscZsaY
**Routine12**	9000	28000	19000	39.58	https://youtu.be/vFCFyKGlAsQ
**Routine13**	19000	55000	36000	75.00	https://youtu.be/Kpp33rjC7L0
**Routine14**	7500	29800	22300	46.46	https://youtu.be/5RhaSvwQL6c
**Routine15**	6800	56600	49800	103.75	https://youtu.be/QtC-EdXwDQ4
**Routine16**	5700	24660	18960	39.50	https://youtu.be/YBIFk1me3hs
**Routine17**	16100	50500	34400	71.66	https://youtu.be/kPlLd-m8xHg
**Routine18**	11500	45400	33900	70.62	https://youtu.be/M-UVxZMgsZI
